# Outcome of Cerebral Venous Thrombosis Requiring Mechanical Ventilation

**DOI:** 10.3390/jcm14092930

**Published:** 2025-04-24

**Authors:** Jayantee Kalita, Prakash C. Pandey, Nagendra B. Gutti, Kuntal K. Das, Sunil Kumar, Varun K. Singh

**Affiliations:** 1 Department of Neurology, Sanjay Gandhi Post Graduate Institute of Medical Sciences, Raebareli Road, Lucknow 226014, Uttar Pradesh, India; drprakashpandey@gmail.com (P.C.P.); drnag1289@gmail.com (N.B.G.); 2 Department of Neurosurgery, Sanjay Gandhi Post Graduate Institute of Medical Sciences, Raebareli Road, Lucknow 226014, Uttar Pradesh, India; drkuntalkantidas@gmail.com; 3 Department of Radiology, Sanjay Gandhi Post Graduate Institute of Medical Sciences, Raebareli Road, Lucknow 226014, Uttar Pradesh, India; anitasunilk@yahoo.co.in; 4 Department of Neurology, Banaras Hindu University, Varanasi 221005, Uttar Pradesh, India; mailurvarun@gmail.com

**Keywords:** stroke, cerebral venous sinus thrombosis, mechanical ventilation, death, outcome

## Abstract

**Background:** Patients with cerebral venous thrombosis (CVT) requiring mechanical ventilation (MV) may have a severe procoagulant state, extensive venous sinus thrombosis, and a worse outcome, but there is a paucity of studies on this topic. We compare the clinical risk factors, radiological findings, and outcomes between CVT patients requiring MV and the non-MV group. **Methods:** Consecutive CVT patients admitted to our service were included. Their clinical details, prothrombotic states and MRI and MRV findings were noted. The patients were admitted to the intensive care unit (ICU) if the Glasgow Coma Scale (GCS) score was below 14 and intubated if arterial blood gas analysis was abnormal. All the patients received heparin followed by an oral anticoagulant. In-hospital death was noted, and functional outcomes at 3 months were assessed using the modified Rankin Scale (mRS). **Results:** Ninety-eight patients with CVT were admitted during the study period; 45 (45.9%) required ICU care, and 18 of them required MV for a median of 6.5 (1–15) days. The MV patients had a shorter duration of illness, a lower GCS score, and protein C deficiency. Twelve (12.2%) patients died: five (27.8%) in the MV, four (14.8%) in the non-MV ICU, and three (5.7%) in the non-MV non-ICU groups. Poor outcomes were 5.5%, 14.8%, and 5.7%, respectively. On Cox regression analysis, the MV had an association with death [adjusted hazard ratio (AHR) 0.40, 95% confidence interval 0.21–0.77; *p* = 0.007] and poor outcome at 3 months (AHR 0.45, 95% CI 0.27–0.76; *p* = 0.003). **Conclusions:** About 18.4% of CVT patients require MV with a mortality of 27.8%. Amongst the survivors, 90.7% of patients have a good outcome at 3 months.

## 1. Introduction

In 2021, stroke was the third leading cause of death worldwide. Out of 11.9 million incident strokes, 65.3% of patients had ischemic, 28.8% had hemorrhagic, and 5.5% had subarachnoid hemorrhage. The majority of deaths occurred during the acute phase [1]. Large multicenter population-based studies have reported the requirement of mechanical ventilation (MV) in 10–15% of stroke patients. The requirement of MV is higher in intracerebral hemorrhage (ICH; 29–30%) compared to ischemic strokes (8%) [2]. Stroke patients requiring MV have high short-term mortality [3,4], withdrawal of care [5,6], and readmission rates [7,8]. The survivors had severe disability [9,10]. A prospective multicenter cohort study including 366 stroke patients requiring MV for at least 24 h revealed poor outcomes in 66.5%, including deaths in 52.2%. The independent predictors of poor outcome were age ≥ 70 years (odds ratio [OR], 2.38 [95% CI, 1.26–4.49]), Charlson Comorbidity Index ≥ 2 (OR, 2.01 [95% CI, 1.16–3.49]), and a Glasgow Coma Scale (GCS) score of less than 8 (OR, 0.56 [95% CI, 0.33–0.94]) [11]. These studies did not include the patients with CVT.

Cerebral venous thrombosis (CVT) is a stroke-like illness and accounts for 0.5–1% of all strokes [12,13,14]. Patients with this condition are younger and have a subacute to chronic presentation with hereditary or acquired prothrombotic states. The association of CVT with the peripartum period has been reported in southeast Asia and its association with oral contraceptives has been reported in western countries [15]. Patients with CVT often present with raised intracranial pressure, and half the patients have seizures and parenchymal lesions in the form of infarction, hemorrhagic infarction, or intracerebral hematoma [16,17,18,19]. There is a paucity of studies evaluating the death and disability of CVT patients requiring MV [20,21]. We hypothesize that the requirement of MV in a patient with CVT is likely to be associated with more severe illness, a severe procoagulant state, more extensive venous sinus thrombosis, and a worse outcome compared to the non-MV group. In this communication, we compare the risk factors, MRI and MRV findings, and outcomes of CVT patients requiring MV compared to those of the non-MV group.

## 2. Material and Methods

This is a retrospective analysis of the CVT patients admitted to a tertiary care teaching hospital between 2010 and February 2022. The Institute Ethics Committee provided ethical approval (PGI/BE/774, 14 October 2015).

### 2.1. Inclusion Criteria

Cerebral venous sinus thrombosis was suspected in a patient presenting with a new-onset headache, seizure, focal neurological deficit, or papilledema. The diagnosis of CVT was confirmed by magnetic resonance venography (MRV). CVT patients with a GCS score of ≤13 were admitted to the ICU.

### 2.2. Exclusion Criteria

Patients with malignancy, rhino-cerebral fungal infection, organ transplantation, or cancer chemo- or radiotherapy; children below 15 years; and those with liver, kidney, or heart failure were excluded.

### 2.3. Clinical Evaluation

The demographic information (age, gender, dietary habit) and clinical details were noted. The duration of illness and the presenting symptoms, including headache, vomiting, seizure, status epilepticus (SE), focal motor deficit, visual loss, diplopia, altered sensorium, and somnolence were recorded. Consciousness was assessed by the Glasgow Coma Scale (GCS). The presence of papilledema and cranial nerve palsy were recorded. Muscle power was classified on a 0–V Medical Research Council (MRC) scale. Muscle tone, tendon reflexes, sensations, and incoordination were noted.

### 2.4. Investigations

Five ml of venous blood were collected in an EDTA vial for complete blood count, homocysteine (Hcy), methyl tetrahydrofolate reductase (MTHFR), HbA1c, and paroxysmal nocturnal hemoglobinuria testing. Blood was collected in a citrate buffer vial for prothrombin time, activated partial thromboplastin time, D-dimer, lupus anticoagulant, protein C and S, antithrombin III, and fibrinogen levels. For serum chemistry, thyroid function tests, autoantibodies, and vitamin B12, blood was collected in a plain vial. Blood counts, hemoglobin, erythrocyte sedimentation rate at the first hour, prothrombin time, activated partial thromboplastin time, blood glucose, blood urea nitrogen, serum creatinine, bilirubin, transaminase, sodium, potassium, calcium, alkaline phosphatase, and albumin were done. A thyroid profile was done in suspected patients.

### 2.5. Risk Factor Evaluation

Female patients, pregnancy, puerperium, and use of oral contraceptive pills were noted. They were evaluated for genetic {factor V Leiden and MTHFR gene mutation, antithrombin III, protein C, and S deficiency} and acquired prothrombotic states [Hcy, antiphospholipid antibody, antinuclear antibody, and anti-double-stranded DNA (anti-ds DNA) antibodies]. Serum folic acid and vitamin B12 were measured in the patients with hyperhomocysteinemia. MTHFR 677C→T gene and Factor V Leiden gene mutations were assessed using polymerase chain reaction. Serum vitamin B12 and folate and plasma homocysteine were measured by the chemiluminescence immunoassay method using a Chemiluminescent Immunoassay System (Immuilte-1000; Siemens Healthcare Diagnostics Technical Services, catalog number: LKVB1, Camberley, UK). Enzyme-linked immunosorbent assay (ELISA) was used for antiphospholipid antibody (Chorus kit, Monteriggioni, Italy) and anti-ds DNA antibody (Chorus kit, Monteriggioni, Italy) and immunofluorescence (Euroimmun kit, Lübeck, Germany). The nephelometry (Siemens kit, Beijing, China) method was used for the measurement of C3 and C4. Antithrombin III (STA Stachrom AT III), protein C (STA-STACLOT PROTEIN C, Stago Diagnostics, NJ, USA), and protein S (STA STACLOT PROTEIN S, Stago Diagnostics, NJ, USA) were assayed using an analyzer (model no. CC32019673, Stago Canada Ltd., Mississauga, ON, Canada). These tests are done routinely in our institute by immunology and genetic departments.

### 2.6. MRI and MR Venography

Cranial magnetic resonance imaging (MRI) and MR venography (MRV) were performed using a 1.5/3T MRI machine (Signa GE Medical System, Wisdom, Chicago, IL, USA). The location and nature of the parenchymal lesion were noted. Contrast MRV was done, and the location and extent of thrombosis of the sinuses were noted. The cerebral venous sinus thrombosis (CVST) score was also calculated [14,22,23,24,25,26].

### 2.7. Mechanical Ventilation

Impending respiratory failure was suspected in the patients with raised intracranial pressure, and the bedside clinical features of raised intracranial pressure, including deterioration in consciousness, pupillary asymmetry, extensor posturing, hyperventilation, and shallow or irregular respiration, were monitored closely. These patients were monitored in the ICU for oxygen saturation and CO_2_ retention. If the arterial blood gas analysis did not normalize with Venti mask oxygen therapy, patients were intubated and mechanically ventilated [27]. We did not do elective intubation due to resource constraints. Mechanical ventilation was done using the Maquet Critical Care AB ventilator (Solna, Sweden) in synchronized intermittent mandatory ventilation (SIMV) mode. The pressure support was kept between 5 and 15 cm of H_2_O for spontaneous breath, and the tidal volume was kept at 5 mL–8 mL/kg for non-spontaneous breath. The weaning protocol was started once the vitals were stable and the patient had regained consciousness. Tracheostomy was done if MV was required for more than 15 days. The duration of MV and occurrence of ventilator-associated pneumonia were noted. Other complications, such as pneumonia, deep vein thrombosis, bedsore, and septicemia, were noted in both the MV, non-MV ICU, and non-MV non-ICU groups. The patients were managed in the neurology ICU by the neurology residents and faculties. In difficult intubation and ventilation, critical care medicine personnel were consulted. The neurosurgeon (KKD) was consulted for hemicraniectomy. One of the faculty (JK) was involved in the management of these patients during the entire period.

### 2.8. Treatment

The patients were treated with low-molecular-weight heparin (enoxaparin 100 unit/kg twice daily) for 10 days. Some patients (who could not afford it financially) received unfractionated heparin (5000 IU intravenously followed by 18 IU/Kg/hour infusion) to keep the activated partial thromboplastin time (APTT) at 2.5 times the control. Thereafter, an anticoagulant (acenocoumarin) was prescribed to maintain the international normalized ratio (INR) at 2–3. The patients with seizures received levetiracetam (500 to 750 mg twice daily) with or without clobazam (5 to 10 mg twice daily). Patients with SE received intravenous lorazepam (0.1 mg/kg~4mg), and if the seizure was not controlled within 10 min, a second dose of lorazepam was introduced. A second-line antiseizure medication was administered to the patients with ongoing convulsion and included levetiracetam (20 mg/kg IV at 100 mg/min) or lacosamide (400 mg at 60 mg/min). Patients were considered to have refractory SE if they failed to respond to second-line antiseizure medication, and for those patients, clobazam and/or lamotrigine were added. Termination of convulsive SE was evaluated clinically, and electroencephalography was done in those with persistent unconsciousness to rule out nonconvulsive status epilepticus. Acetazolamide (250 mg thrice daily) with or without a bolus dose of 100 mL of 20% mannitol was prescribed to the patients with clinical features of raised intracranial pressure. The patients not responding to conventional antiedema treatment underwent hemicraniectomy.

### 2.9. Outcomes

The outcome was defined using the modified Rankin Scale (mRS). In-hospital death was noted. The surviving patients were followed up on at 3 months, and their outcomes were categorized as good (mRS 0–2) or poor (mRS 3, 4, 5) [25].

### 2.10. Statistical Analysis

The normalcy of data was verified by the Wilk–Shapiro test. The demographic, clinical, and risk factors; MRI and MRV findings; duration of hospitalization; complications; and outcomes between the MV, non-MV ICU, and non-MV non-ICU groups were compared by a chi-square test if categorical and a one-way analysis of variance if there was a continuous variable. The association of variables with death and poor outcome was also evaluated using a parametric or non-parametric test. The effect size of MV on death and poor outcome was calculated. Cox regression analysis was done for the evaluation of the hazard ratio of MV in determining death and 3-month poor outcome. The statistical analysis was done using SPSS 20 version software. A variable was considered significant if the exact two-sided *p* value was <0.05.

## 3. Results

### 3.1. Clinical and Risk Factors

Ninety-eight patients with CVT were admitted during the study period, and 45 (45.9%) patients required ICU admission. Eighteen out of 45 (40%) ICU patients required MV. Their mean age was 32.09 (SD 12.75) years, and 43 (43.9%) were females. They were admitted after a median of 7 days (range 1–62 days) from their initial symptoms. The presenting symptoms were headache in 90 patients (91.8%), vomiting in 61 patients (62.2%), focal neurological deficit in 62 (63.3%), and seizures in in 69 (70.4%) patients. Twenty-nine (29.6%) patients had status epilepticus. Risk factors of CVT could be identified in 71 (72.4%) patients; protein S deficiency and hyperhomocysteinemia were the most common, followed by MTHFR mutation. The details are presented in Table 1. Thyroid hormones were assayed in 56 patients; the median thyroid-stimulating hormone (TSH) level was 2.33 (0.25–8.74 IU/mL). However, none had abnormal levels of tri- or tetraiodothyronine requiring treatment.

### 3.2. MRI and MRV Findings

Cranial MRI revealed a parenchymal lesion in 85 (86.7%) pale infarctions in 16, and hemorrhagic infarctions in 69 patients. MR venography revealed involvement of the superficial sinuses in 84 (85.7%), deep in 4 (4.1%), and both in 10 (1%) patients. The details are presented in Table 2.

### 3.3. Comparison of MV and Non-MV Patients

The MV patients had lower GCS scores compared to non-MV patients (8.39 ± 3.22 vs. 12.95 ± 3.23; *p* = 0.001). Protein C deficiency was also more frequently associated with the MV group (46.2% vs. 12.5%; *p* = 0.03). The other clinical and risk factors were comparable, between MV and non-MV groups. MRI lesions and MRV findings were also comparable, including the number of sinuses involved. The details are presented in the Supplementary Table S1.

### 3.4. Comparison of MV and Non-MV ICU Patients

Eighteen out of 45 (40%) CVT patients in the ICU required MV within 1–3 days of hospitalization. The reason for intubation was raised intracranial pressure in all, and eight patients also had SE.

#### 3.4.1. Comparison of Clinical Parameters

The clinical variables, including age, gender, duration of illness, SE, focal motor weakness, and the GCS score, were not significantly different between the two groups (Table 1).

#### 3.4.2. Comparison of MRV and MRI Findings

**Figure 1:**
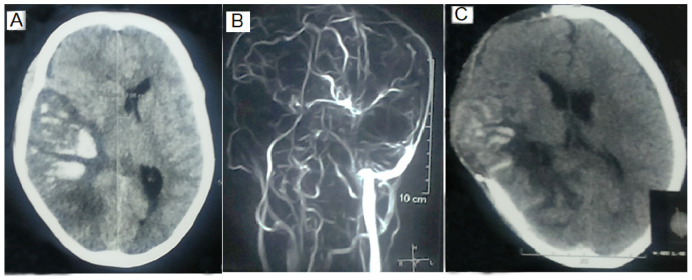
CT scan and MR venography of a patient with cerebral venous thrombosis showing hemorrhagic infarction in the parieto-occipital area with effacement of sulci and ventricle and midline shift (**A**). On MR venography, there was thrombosis of both the superficial and deep systems (**B**). The patient underwent hemicraniectomy on the third day of hospitalization, and the postoperative CT scan revealed improvement in mass effect as evidenced by the reappearance of the lateral ventricle and reduced midline shift (**C**).

The location and extent of thrombosis did not determine the requirement of MV. The number of venous sinus thrombosis cases in MV and non-MV ICU patients was comparable (1.33 ± 0.48 vs. 1.26 ± 0.45; *p* = 0.60). Superior sagittal sinus was the commonest site of thrombosis (n = 31, 68.9%), followed by transverse (n = 29, 64.4%) and sigmoid (n = 19, 42.2%), which were also comparable between the two groups (Table 2, Figure 1). The presence of a parenchymal lesion (16 vs. 23; *p* = 1.00) and the frequency of hemorrhagic (15 vs. 17) and pale infarctions (1 vs. 6) were also comparable (*p* = 0.21). The volume of parenchymal lesion was measured in 40 out of 45 patients admitted to ICU (17 in the MV group and 23 in the non-MV ICU group) and was comparable between MV and non-MV ICU patients (43.94 ± 77.28 mL vs. 26.80 ± 16.82 mL; *p* = 0.30).

#### 3.4.3. Comparison of Risk Factors

Protein C deficiency was more frequent in the MV group (46% vs. 11.8%; *p* = 0.04), whereas the frequency of the remaining risk factors was comparable between the groups. The number of risk factors in each patient ranged between none and five, and 10 patients had more than two risk factors. The number of risk factors was insignificantly higher in the non-MV group (1.25 ± 0.58 vs. 1.67 ± 6.76; *p* = 0.06). The details are presented in Table 1.

### 3.5. Outcomes

**Figure 2:**
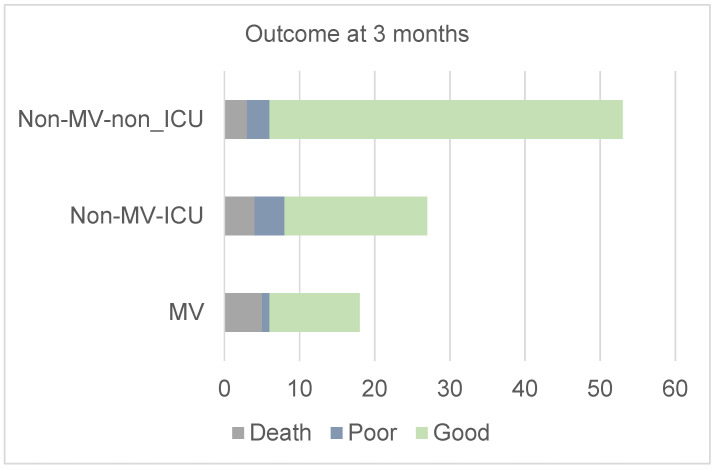
Number of patients having death, poor, and good outcomes in the mechanical ventilation (MV), non-MV ICU (intensive care unit), and non-MV non-ICU groups.

Twelve (12.2%) patients died in the hospital: five (27.8%) in the MV group, four (14.8%) in the non-MV ICU group, and three (5.7%) in the non-MV non-ICU group. The cause of death was intracranial pressure leading to trans-tentorial herniation. Two of these patients had decompressive surgery: one died, and the other survived. The requirement of MV had an effect size of 2.2 times for defining death compared to non-MV ICU patients. Amongst the survivors, 78 (79.6%) patients had a good outcome, and 8 (8.2%) had a poor outcome, which was not significantly different amongst the MV, non-MV ICU, and the non-MV non-ICU groups (*p* = 0.08; Figure 2).

**Figure 3:**
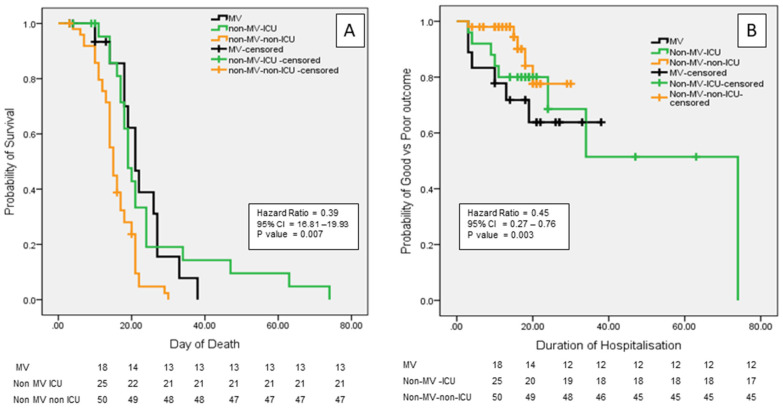
Kaplan–Meier curves. (**A**) The probability of survival was significantly lower in the patients requiring mechanical ventilation (MV) compared to the non-MV ICU (intensive care unit) and non-MV non-ICU groups. (**B**) The probability of poor outcome was higher in the patients requiring MV.

On univariate analysis, death was associated with GCS score (*p* = 0.01), acute in presentation (*p* = 0.03), and requirement of MV (*p* = 0.04). The other clinical, MRI, and MRV findings and CVT risk factors were not associated with death (Table 3). On Cox regression analysis, the requirement of MV had a significant association with death [adjusted hazard ratio (AHR) 0.40, 95% confidence interval 0.21–0.77; *p* = 0.007; Figure 3A]. The 3-month poor outcome was associated with GCS score (*p* = 0.001), acute presentation (*p* = 0.02), and focal motor deficit (*p* = 0.03) on univariate analysis. The poor outcome was not significantly different amongst the MV, non-MV ICU, and non-MV non-ICU groups (*p* = 0.09). The details are presented in Table 4. On Cox regression analysis, the requirement of MV had a significant association with poor outcome at 3 months (AHR 0.45, 95% CI 0.27–0.76; *p* = 0.003; Figure 3B).

### 3.6. Complications

The duration of MV ranged between 1 and 15 (median 6.5) days. None of the patients required a tracheostomy. One patient developed ventilator-associated pneumonia in the MV group, and he was treated with ceftriaxone and clindamycin. This patient had good recovery at 3 months. Another patient developed septicemia and responded to ceftriaxone and levofloxacin. Two patients in the non-MV group also developed septicemia, and they were also treated with ceftriaxone and levofloxacin. These patients had a good recovery at 3 months.

## 4. Discussion

About 45.9% of CVT patients required ICU admission, and 40% of them required MV for a median duration of 6 days. The patients in the MV group had an acute presentation with a lower GCS score and a protein C deficiency state. None of the other clinical, MRI, or MRV findings or other CVT risk factors were associated with MV requirement. Death occurred in 27.8% of patients in the MV group with an AHR of 0.04. Poor outcome at 3 months was also associated with the requirement of MV. The effect size of death was 2.2 in the MV group compared to the non-MV ICU group, although MV and non-MV ICU patients did not have a significant difference in death and poor outcome. There is only one previous study by Soyer et al. on the outcome of CVT patients admitted to the ICU. In that study, 37 out of 41 (90%) patients required MV: 10 (27%) patients died, and 27 survivors improved to an mRS score of 2–4 at 3 months. A focal deficit and the type and location of the stroke were not associated with death, but raised intracranial pressure was associated with death [20]. Large population-based studies have reported the requirement of MV in 10–15% of patients with acute stroke, 2.9–30% of patients with subarachnoid hemorrhage and intracerebral hemorrhage, and 8% of patients with ischemic stroke [3,4,5,6,7,8,9,10]. In a study on 31,300 ischemic strokes, the requirement of MV had a hazard ratio of 5.6 for 30 days of mortality [28]. In the study by Steiner et al., the 1-year survival rate was 33.1%, and 52% died in the neurological intensive care unit. The independent predictors of death at 2 months were age greater than 65 years (*p* = 0.03), reason for intubation (coma or respiratory failure), and a GCS score less than 10 [7]. The majority of previous studies intubated the stroke patients based on the GCS score. Death in MV patients depends on the reason for intubation: intubation due to respiratory failure or coma had a higher mortality than those requiring intubation due to seizures [2]. We, however, intubated if there was an arterial blood abnormality due to SE or raised intracranial pressure. Montmollin el at. have reported survival of stroke patients requiring MV. The mortality at one month was the highest if the reason for intubation was cardiac arrest (100%), followed by altered mental status (72.8%), acute renal failure (55.8%), seizure (47.1%), and elective intubation (8.3%) [2]. In our study, 27.8% of patients on MV died, although all had altered mental status, seizure/status epilepticus, and respiratory failure.

The requirement of MV in an earlier cohort of CVT was 13.6%, with an overall death in 2% [28]. The mortality rate in patients with CVT ranges between 3.4% and 9.9%. More than 80% of survivors have a good outcome [17,19,25]. In the present study, the in-hospital overall mortality was 12.2% and is comparable with the reported studies. This is much lower than the mortality for ischemic and hemorrhagic strokes, in whom in-hospital mortality ranges between 53% and 57% [3,4,5], and one-year mortality ranges between 60% and 92% [6,7,8,9,10]. In the present study, none of the patients died after discharge, and the surviving patients progressively improved and had a good outcome at 3 months in 66.7%, 70.4%, and 88.7% of cases in MV, non-MV ICU, and non-MV non-ICU patients, respectively.

The predictors of death in ischemic and hemorrhagic strokes are age, comorbidities, GCS score, NIHSS score, size of the stroke, midline shift, herniation, raised intracranial pressure, and biomarkers of inflammation [29,30,31,32]. In the present study, a shorter duration of illness and a lower GCS score were associated with death. In the International Study on Cerebral Vein and Dural Sinus Thrombosis, 624 adult patients with CVT were followed up on for a median of 16 months, of whom 8.3% of patients died and 81.6% had a good recovery (mRS ≤ 2). Upon multivariate analysis, the predictors of death or dependence were age > 37 years, male sex, coma, mental status disorder, hemorrhagic changes on admission CT scan, thrombosis of the deep cerebral venous system, central nervous system infection, and cancer [17]. In our earlier report, none of the clinical risk factors, MRI, or MRV findings were related to death or poor outcome [33]. The extent of thrombosis as assessed by CVST score and the number of parenchymal lesions were also not related to death or 6-month outcomes [24,33].

In the present study, the proportion of SE patients was comparable between the MV and the non-MV ICU groups as well as between the death and the survivor groups. In a study, the death and disability of CVT patients were not significantly different between SE, self-limiting seizure, and no-seizure groups. At 6 months, 84% of patients with SE, 92.3% with self-limiting seizures, and 94.8% in the no-seizure group had a good recovery [26]. The lower mortality and a better long-term outcome of CVT patients may be due to venous congestion rather than a lack of blood flow in thrombotic stroke. Both ischemic stroke and CVT may have recanalization of blood vessels [22,31,34,35,36,37,38,39]. In a meta-analysis including 694 patients, repeat MRV after a variable time of the event revealed recanalization in 85% of patients [37,39]. Complete resolution of the parenchymal lesion at follow-up has also been reported in 13.2% of patients, and they recovered without sequelae [40,41,42]. In the present study, pneumonia and septicemia occurred in very few patients, which were comparable in the MV and the non-MV ICH groups. This may be due to the short duration of MV, younger age, and non-diabetic status.

In our study, the protein C deficiency state was associated with MV. Protein C deficiency predisposes individuals to a hypercoagulable state, increasing the risk of thrombotic events such as deep vein thrombosis and pulmonary embolism. Pulmonary thromboembolism can lead to significant respiratory compromise, potentially requiring mechanical ventilation. There are a few case reports of patients with concurrent deficiencies of protein C, protein S, and antithrombin III who developed massive pulmonary embolism necessitating mechanical ventilation [43]. The other prothrombotic conditions in CVT may also cause pulmonary embolism. In our cohort of CVT patients, the admission blood pressure, chest radiograph, and ECG were normal. However, we did not perform pulmonary angiography to rule out pulmonary arterial or venous thrombosis in them. Thromboembolic stroke is associated with hypo- or hyperthyroidism. Hypothyroidism is associated with hyperlipidemia and atherosclerosis, and hyperthyroidism is associated with cardiac arrhythmia, and these are potential risk factors for stroke. Raho et al. have reported an association between hypothyroidism and CVT [44]. Thirteen patients in our cohort had a higher TSH level but did not require treatment because their TSH level was below 10 iu/L, and all had normal T3 and T4 levels.

## 5. Limitations

This is a retrospective single-center study based on a small sample size and carried out in a tertiary care center. Cerebral venous thrombosis is a rare form of stroke-like illness; hence, a multicenter prospective study is desirable.

## 6. Conclusions

Mechanical ventilation was required in 18.4% of patients with CVT and in 40% requiring ICU admission. The overall one-month mortality of CVT patients was 12.2%, with a 20% mortality in ICU patients and 27.8% in MV patients. The majority of survivors had a good outcome at 3 months. The requirement of MV was an independent predictor of death and poor outcome.

## Figures and Tables

**Table 1:** Comparison of clinical and risk factors between the patients requiring mechanical ventilation (MV), non-MV ICU (intensive care unit) patients, and non-MV non-ICU patients with cerebral venous thrombosis.

	Total (n = 98)	MV (n = 18)	Non-MV ICU (n = 27)	Non-MV Non-ICU (n = 53)	*p*
Age (yrs)	32.09 ± 12.75	29.44 ± 11.91	36.67 ± 13.31	30.66 ± 12.36	0.08
Females	43 (43.9%)	6 (33.3%)	16 (59.3%)	21 (39.6%)	0.43
Duration of illness (Days)	25.92 ± 76.04	12.66 ± 15.02	10.67 ± 11.84	38.21 ± 101.53	0.22
Onset					0.02
Acute (≤2 days)	8 (8.2%)	4 (22.2%)	3 (11.1%)	1 (1.9%)	
Sub-acute (3–30 days)	76 (77.6%)	12 (65.7%)	23 (85.2%)	41 (77.4%)	
Chronic > 30 days	14 (14.3%)	2 (11.1%)	1 (3.7%)	11 (20.8%)	
Seizures	69 (70.4%)	10 (55.6%)	18 (66.7%)	41 (77.4%)	0.20
Status epilepticus	29 (29.6%)	8 (44.4%)	9 (33.3%)	12 (22.6%)	0.25
Focal deficit	62 (63.3%)	14 (77.8%)	19 (70.4%)	29 (54.7%)	0.14
GCS score	12.11 ± 3.67	8.39 ± 3.22	9.11 ± 2.91	14.91 ± 0.30	0.001
Risk factor Hereditary prothrombotic states					
MTHFR					0.73
CC	40 (67.8%)	3 (75%)	13 (76.5%)	24 (64.9%)	
CT	18 (30.5%)	1 (25%)	4 (23.5%)	12 (32.4%)	
TT	1 (1.7%)	0 (0%)	0 (0%)	1 (2.7%)	
Antithrombin III					0.37
Normal	48 (90.6%)	9 (81.8%)	11 (100%)	28 (90.3%)	
Deficient	5 (9.4%)	2 (18.2%)	0 (0%)	3 (9.7%)	
Protein S					0.11
Normal	37 (52.1%)	10 (71.4%)	11 (61.1%)	16 (41.0%)	
Deficient	34 (47.8%)	4 (28.6%)	7 (38.9%)	23 (59.0%)	
Protein C					0.05
Normal	56 (77.8%)	7 (53.8%)	17 (89.5%)	32 (77.8%)	
Deficient	16 (22.2%)	6 (46.2%)	2 (10.5%)	10 (20.0%)	
Acquired prothrombotic state					0.50
Homocysteine µmol/L					
<15	38 (52.0%)	4 (66.7.2%)	13 (59.1%)	21 (46.7%)	
≥15	35 (47.9%)	2 (33.3%)	9 (40.9%)	24 (53.3%)	
APLA syndrome					0.52
Yes	13 (13.3%)	3 (16.7%)	5 (18.5%)	5 (9.4%)	
No	85 (86.7%)	15 (83.3%)	22 (81.5%)	48 (90.6%)	
Vitamin B12 pg/mL					0.29
<200	28 (34.1%)	3 (25%)	8 (36.4%)	17 (35.4%)	
200–500	21 (25.6%)	1 (8.3%)	5 (22.7%)	15 (31.2%)	
>500	33 (40.2%)	8 (66.7%)	9 (40.9%)	16 (33.3%)	
Folic acid ng/mL					0.29
<3.5	25 (25.5%)	3 (16.7%)	5 (18.5%)	17 (32.1%)	
>3.5	73 (74.5%)	15 (83.3%)	22 (81.5%)	36 (67.9%)	
Female Specific risk					0.37
Puerperium					
Yes	12 (27.9%)	2 (33.3%)	5 (31.3%)	15 (48.4%)	
No	31 (72.1%)	4 (66.4%)	11 (68.8%)	16 (51.6%)	
Oral contraceptives pill					0.40
Yes	5 (11.6%)	1 (16.7%)	1 (6.3%)	3 (14.3%)	
No	38 (88.4%)	5 (83.3%)	15 (93.7%)	18 (85.7%)	
Number of risk factors	1.42 ± 1.25	1.38 ± 1.03	1.59 ± 1.45	1.36 ± 1.23	0.73
No risk factor	27 (27.6%)	3 (16.7%)	7 (25.9%)	17 (32.1%)	0.51
Outcome at 3 months					0.05
Good	78 (79.6%)	12 (66.7%)	19 (70.4%)	47 (88.7%)	
Poor	8 (8.2%)	1 (5.5%)	4 (14.8%)	3 (5.7%)	
Death	12 (12.2%)	5 (27.8%)	4 (14.8%)	3 (5.7%)	

APLA = antiphospholipid antibody syndrome, GCS = Glasgow Coma Scale, MTHFR = methyl tetrahydrofolate reductase.

**Table 2:** Comparison of MRI and MR venography (MRV) findings between the patients requiring mechanical ventilation (MV), non-MV ICU (intensive care unit) patients, and non-MV non-ICU patients with cerebral venous thrombosis.

Parameter	All n = 98	MV n = 18	Non-MV ICU n = 27	Non-MV Non-ICU n = 53	*p*
Parenchymal lesion on MRI	85	16 (88.9%)	23 (85.2%)	46 (86.8%)	0.58
Infarct	16	1 (5.6%)	6 (22.2%)	9 (16.9%)	
Hemorrhagic	69	15 83.3.%)	17 (62.9%)	37 (69.8%)	
Thrombosis on MRV					0.95
Superficial system	84	15 (83.3%)	22 (81.5%)	47 (88.7%)	
Deep system	4	1 (5.6%)	1 (3.7%)	2 (3.8%)	
Both	10	2 (11.1%)	4 (14.8%)	4 (75.5%)	
Superior sagittal sinus	68	13 (72.2%)	18 (66.7%)	37 (69.8%)	0.91
Inferior sagittal sinus	4	0 (0%)	2 (7.4%)	2 (3.8%)	0.55
Transverse sinus	63	13 (72.2%)	16 (59.2%)	34 (64.1%)	0.69
Sigmoid sinus	46	9 (50.0%)	10 (37.0%)	27 (50.9%)	0.52
Number of sinuses involved	1.63 ± 0.74	1.33 ± 0.48	1.63 ± 0.69	1.73 ± 0.81	0.13

**Table 3:** Predictors of death of the patients with cerebral venous thrombosis with univariate analysis.

	Death N = 12	Survived N = 86	*p*
Age (yrs)	30.25 ± 12.17	32.35 ± 12.87	0.59
Gender (Male/Female)	8 (66.7%)/4 (33.3%)	47 (54.7%)/39 (45.3%)	0.42
Duration (days)	9.00 ± 8.58	28.29 ± 80.89	0.43
Acute	3 (25.0%)	5 (5.8%)	0.03
Sub	9 (75.0%)	67 (77.9%)	
chronic	0 (0%)	14 (16.3%)	
Status epilepticus	3 (25.0%)	27 (31.4%)	0.75
Motor deficit	10 (83.3%)	52 (60.5%)	0.20
Number of risk factors	0.83 ± 0.71	1.51 ± 1.29	0.08
MRI parenchymal lesion	12 (100%)	73 (84.9%)	0.05
MRV			0.38
Deep	0 (0.0%)	4 (4.7%)	
Superficial	10 (83.3%)	74 (86.0%)	
Both	2 (16.7%)	8 (9.3%)	
GCS score	8.92 ± 4.29	12.56 ± 3.37	0.01
Requirement of mechanical ventilation (MV)/Intensive care unit (ICU)			0.04
MV	5 (27.8%)	13 (72.2%)	
Non-MV ICU	4 (14.8%)	23 (85.2%)	
Non-MV non-ICU	3 (5.7%)	50 (94.3%)	

GCS = Glasgow Coma Scale, ICU = intensive care unit, MV = mechanical ventilation, MRV = magnetic resonance venography.

**Table 4:** Predictors of 3-month outcomes of the patients with cerebral venous thrombosis.

	Poor N = 20	Good N = 78	*p*
Age (yrs)	35.00 ± 14.49	31.39 ± 12.28	0.27
Gender (Male/Female)	12 (63.1%)/7 (36.8%)	43 (54.4%)/36 (45.5%)	0.60
Duration (days)	12.79 ± 14.53	29.08 ± 84.21	0.40
Acute	4 (21.1%)	4 (5.0%)	0.02
Sub acute chronic	14 (73.7%) 1 (5.2%)	62 (78.5%) 13 (16.5%)	
Status epilepticus	7 (23.3%)	23 (76.7%)	0.58
Motor deficit	16 (25.8%)	46 (74.2%)	0.04
Number of risk factors	1.00 ± 1.00	1.53 ± 1.29	0.10
MRI parenchymal lesion	18 (21.2%)	67 (78.8%)	0.30
MRV			0.05
Deep	0 (0.0%)	4 (5.1%)	
Superficial	14 (73.7%)	70 (88.6%)	
Both	5 (26.3%)	5 (6.3%)	
GCS score	6.89 ± 2.62	9.31 ± 2.95	0.03
Requirement of mechanical ventilation (MV)/Intensive care unit (ICU)			0.09
Mechanical ventilation	6 (33.3%)	12 (66.7%)	
Non-MV ICU	8 (29.6%)	19 (70.4%)	
Non-MV Non-ICU	5 (9.4%)	48 (90.6%)	

GCS = Glasgow Coma Scale, ICU = intensive care unit, MV = mechanical ventilation, MRV = magnetic resonance venography.

## Data Availability

Data will be available upon reasonable request from the corresponding author.

## Supplementary Materials

The following supporting information can be downloaded at: https://www.mdpi.com/article/10.3390/jcm14092930/s1, Supplementary Table S1. Comparison clinical and risk factors between the patients requiring mechanical ventilation (MV) compared with others cerebral venous thrombosis patients in intensive care unit. Supplementary Table S2. Comparison of MRI and MRV findings in cerebral venous thrombosis patients in intensive care requiring mechanical ventilation (MV) compared to others.
