# Supplementary material for: Outcome of Cerebral Venous Thrombosis Requiring Mechanical Ventilation
Source: J Clin Med. 2025 Apr 24;14(9):2930. doi: 10.3390/jcm14092930 (PMC12072781; doi:10.3390/jcm14092930)
Supplement: Supplementary file 1 [file jcm-14-02930-s001.zip › jcm-3487424-supplementary.pdf]

Supplementary Table 1. Comparison clinical and risk factors between the patients requiring mechanical ventilation (MV) compared with others cerebral venous thrombosis patients in intensive care unit.

	**Total (n=98)**	**MV (n=18)**	**Others (n=80)**	**P**
**Age (yrs)**	32.09±12.75	29.44±11.91	32.69±12.92	0.33
**Females**	43(43.9%)	6(33.3%)	37(46.25%)	0.43
**Duration of illness (Days)**	25.92±76.04	12.66±15.02	10.67±11.87	0.62
**Onset**<br>**Acute (≤ 2 days)**<br>**Sub-acute (3-30 days)**<br>**Chronic >30 days**	 8(8.2%)<br>76(77.6%)<br>14(14.3%)	 4(22.2%)<br>12(65.7%)<br>2(11.1%)	 4(5.0%)<br>64(80.0%)<br>12(15.0%)	0.07
**Seizures**	69(70.4%)	10(55.6%)	53(66.3%)	1.00
**Status epilepticus**	29(29.6%)	8(44.4%)	21(26.3%)	0.25
**Focal deficit**	62(63.3%)	14(77.8%)	48(60.0%)	0.18
**GCS score**	8.08±3.02	8.39±3.22	12.95±3.23	0.001
**Risk factor**<br>**Hereditary prothrombotic states**				
**MTHFR**<br>**CC**<br>**CT**<br>**TT**	 40(67.8%)<br>18(30.5%)<br>1(1.7%)	 3(75%)<br>1(25%)<br>0(0%)	 37(46.3%)<br>17(21.3%)<br>1(2.7%)	1.00
**Antithrombin III**<br>**Normal**<br>**Deficient**	 48(90.6%)<br>5(9.4%)	 9(81.8%)<br>2(18.2%)	 39(48.8%)<br>3(3.8%)	0.57
**Protein S**<br>**Normal**<br>**Deficient**	 37(52.1%)<br>34(47.8%)	 10(71.4%)<br>4(28.6%)	 27(33.8%)<br>30(37.5%)	0.14
**Protein C**<br>**Normal**<br>**Deficient**	 56(77.8%)<br>16(22.2%)	 7(53.8%)<br>6(46.2%)	 49(61.3%)<br>10(12.5%)	0.03
**Acquired prothrombotic state**<br>**Homocysteine µmol/l**<br>**<15**<br>**≥15**	 <br>38(52.1%)<br>35(47.8%)	 <br>4(66.7%)<br>2(33.3%)	 <br>34(42.5%)<br>33(41.3%)	0.67
**APLA syndrome**<br>**Yes**<br>**No**	 13(13.3%)<br>85(86.7%)	 3(16.7%)<br>15(83.3%)	 10(12.5%)<br>79-(92.9%)	0.66
**Vitamin B12 pg/ml**<br>**<200**<br>**200-500**<br>**>500**	 28(34.1%)<br>21(25.6%)<br>33(40.2%)	 3(25%)<br>1(8.3%)<br>8(66.7%)	 25(31.3%)<br>20(25.0%)<br>25(31.3%)	0.79
**Folic acid ng/ml**<br>**<3.5**<br>**≥3.5**	 25(25.5%)<br>73(74.5%)	 3(16.7%)<br>15(83.7%)	 22(27.5%)<br>67(83.8%)	0.39
**Female Specific risk**<br>**Puerperium**				

**Yes**<br>**No**	12(27.9%)<br>31(72.1%)	2(33.3%)<br>4(66.4%)	10(12.5%)<br>27(33.8%)	0.57
**Oral contraceptives pill**<br>**Yes**<br>**No**	 5(11.6%)<br>38(88.4%)	 1(16.7%)<br>5(83.3%)	 4(5.0%)<br>33(41.3%)	0.48
**Number of risk factor**	1.42±1.25	1.25±.5	1.43±1.30	0.40
**No risk factor**	27 (27.6%)	1(20.0%)	24(30.0%)	0.38
**Outcome at 3 months**<br>**Good**<br>**Poor**<br>**Death**	 78 (79.6%)<br>8 (8.2%)<br>12 (12.2%)	 12 (66.7%)<br>1 (5.5%)<br>5 (27.8%)	 66(82.5%)<br>7(8.7%)<br>7(8.7%)	0.08

Supplementary Table 2. Comparison of MRI and MRV findings in cerebral venous thrombosis patients in intensive care requiring mechanical ventilation (MV) compared to others .

Parameter	All<br>n=98	MV<br>n=18	Others<br>n= 80	P
**Parenchymal lesion on MRI**<br>**Infarct**<br>**Hemorrhagic**	85<br>16<br>69	16(18.8%)<br>1(6.3%)<br>15(21.7%)	69(81.2%)<br>15((93.8%)<br>54(78.3%)	0.33
**Thrombosis on MRV**<br>**Superficial system**<br>**Deep system**<br>**Both**	 84<br>4<br>10	 15(17.9%)<br>1(25.0%)<br>2(20.0%)	 69(86.3%)<br>3(75%)<br>8(80.0%)	1.00
**Superior saggital sinus**	68	13(19.1%)	55(80.9%)	0.79
**Inferior saggital sinus**	4	0(0%)	4(100%)	0.59
**Transverse sinus**	63	13(20.6%)	50((79.4%)	0.31
**Sigmoid sinus**	46	9(19.6%)	37(80.4%)	0.48
**Number of sinus involved**	1.63±0.74	1.33±0.48	1.7±0.77`	0.06
